# Anaesthesia for In Vitro Fertilisation

**Published:** 2009-08

**Authors:** Divya Jain, Amit Kohli, Lalit Gupta, Poonam Bhadoria, Raktima Anand

**Affiliations:** 1 Assistant Professor, Department of Anaesthesiology and Intensive Care, Maulana Azad Medical College, New Delhi; 2,3 Senior Resident, Department of Anaesthesiology and Intensive Care, Maulana Azad Medical College, New Delhi; 4 Professor, Department of Anaesthesiology and Intensive Care, Maulana Azad Medical College, New Delhi; 5 Director Professor and Head, Department of Anaesthesiology and Intensive Care, Maulana Azad Medical College, New Delhi

**Keywords:** In vitro fertillization, Monitored anaesthesia, General anaesthesia, Regional anaesthesia

## Abstract

**Summary:**

In vitro fertilization is an upcoming speciality. Anaesthesia during assisted reproductive technique is generally required during oocyte retrieval, which forms one of the fundamental steps during the entire procedure. Till date variety of techniques like conscious sedation, general anaesthesia and regional anaesthesia has been tried with none being superior to the other. However irrespective of the technique the key point of anaesthesia for in vitro fertilization is to provide the anaesthetic exposure for least duration so as to avoid its detrimental effects on the embryo cleavage and fertilization.

## Introduction

In-vitro fertilization (IVF) started 30 years back when Lesley and John Brown, a young couple from Bristol were unable to conceive for 9 years. Lesley had blocked Fallopian tubes. On 10^th^ Nov 1977, Lesley underwent the very experimental in-vitro fertilization by Dr: Patrick Steptor. Finally, on 25^th^ July 1978 LOUISE JOY BROWN, the 1^st^ successful test tube baby was born. Since then there has been continuous refinement in the fertility drug protocols and the techniques to retrieve eggs. As a result, IVF success rates began to climb slowly reaching 25-30%^1^.

## What is In-Vitro Fertilization?

In-Vitro Fertilization is a broad term for the technique of ultrasound directed Oocyte retrieval (UDOR) or Trans Vaginal Follicle Aspiration (TVFA) and fertilization in the laboratory with transfer of embryos back into the uterus.

Broadly speaking IVF involves the following steps

1. Ovarian stimulation
2. Egg collection
3. Sperm processing &
4. Fertilization & embryo transfer

## Discussion

### Role of Anaesthesiologist

1980's witnessed a drastic change from the use of laparoscope to vaginal ultrasound probe for egg retrieval. Although this technique of using Vaginal ultra-sound probe is less invasive and associated with higher pregnancy rates, it forms one of the most stressful and painful components of the entire assisted reproductive treatment^2^,^3^

**Pain** during oocyte retrieval is caused by the puncture of the vaginal skin and ovarian capsule by the aspirating needle as well as manipulation within the ovary during the entire procedure^4^. Here it becomes customary for the anaesthetist to provide adequate pain relief to immobilise the patient and eliminate the danger of piercing any vessel during the process of oocyte retrieval. The ideal pain relief during oocyte retrieval should be effective and safe, easy to administer and monitor, short acting and readily reversible with a few side effects^5^‐^8^.

**Coexisting illness-**Patients presenting in the IVF clinic needs to be investigated for any co morbid illnesses. In India tuberculosis is the most important cause of infertility, so we need to know the drug interactions of anti tubercular drugs with the anaesthetic agents. These patients are generally kept on aspirin or heparin so as to prevent the hypercoaguable state occurring as a result of gonadotrophic injections. Aspirin should ideally be stopped 3 days prior to egg retrieval procedure. In case our patient is on heparin, we need to know the Activated prothrombin time.

Thyroid can also be a cause of infertility so it becomes mandatory to assess the thyroid function tests and take appropriate anaesthetic precautions.

Some of the patients might be receiving treatment forpsychomotor disorders like depression and are on anti depression drugs like Selective serotonin reuptake inhibitors(SSRI), tricyclins or drugs like tragadone, bupropion. It is therefore important to adjust the dosages of anaesthetic agents especially narcotics accordingly.

**Anxiety** – Another major challenge for the anaesthetistis to allay the anxiety. The patients presenting in the IVF clinic are under high degree of social and psychological stress. Majority of them are in late thirties, and the immense family pressure makes them more susceptible to psychomotorillness like depression and psychosis. Moreover this problem is further aggravated by the hormonal manipulation occuring during in vitro fertilization.

Thus it becomes important to provide them with a comfortable environment so as to extract complete medical and pharmacological history. Nowadays many upcoming IVF centres have a provision for isolated rooms for the pre-anaesthetic checkups. One must remember that proper preoperative counseling is very important in allaying anxiety in such patients.

## Types of Anaesthesia

Presently anaesthesia for assisted reproductive tecnique is emerging as a speciality in itself. Patients presenting for IVF can have varied causes for infertility like pelvic inflammatory disease due to tuberculosis, chlamydial infection, history of previous pelvic surgery, tubal blockage or endometriosis. Therefore the patients undergoing this treatment are thoroughly evaluated for the cause of infertility and appropriate treatment instituted. A thorough pre anaesthetic evaluation is required to identify any comorbid illness.

## There are many options available to the anaesthesiologist:

1. Monitored sedation with/without local anaesthesia
2. General Anaesthesia
3. Regional Anaesthesia

A survey conducted by Bokhari et al in U.K showed the use of sedation in 46% of the centres, general anaesthesia in 28%, regional anaesthesia with sedation in 12% while a cocktail regime was followed by the rest 14%^9^.

### Monitored anaesthesia care

Monitored anaesthesia is relatively easy to deliver, drugs are well tolerated and best suited in day care settings. However, it has its own risks of cardiac, respiratory and anaphylactic complications.

- In USA, 95% of the programs use conscious sedation as a part of monitored anaesthesia care^10^
- In UK, 84% of the centres now use sedation^11^

Monitored anaesthesia technique with remifentanil resulted in a higher pregnancy rate than GA with alfentanil+ propofol or isoflurane + propofol for maintenance^12^.

Hadimioglo etal had studied various combination of sedation regimens for oocyte retrieval. and found no significant difference between propofol +fentanyl, midazolam+fentanyl and propofol+fentanyl in the recovery characteristics^13^. Midazolam was found to be safe for sedation in oocyte retrieval^14^,^15^.

### General Anaesthesia

Invariably allanaesthetic agents being used in general anaesthesia have been detected in follicular fluid, raising concerns regarding their use. However, with recent studies documenting the safe use of the agents, balanced anaesthesia with N_2_O and opioids can be an option for anaesthesiologists. Hammadeh etal in 1999, showed a higher retrieval of oocytes with remifentanil + propofol or isoflurane based general anaesthesia than with sedation with midazolam, diazepam or propofol^16^. This could be attributed to the increased comfort level of both the gynaecologist and the patient. With a relaxed uterus, it becomes easier for the gynaecologist to aspirate even the small ovarian follicles, unlike sedation where a contracted myometrium fibrils pose a hinderence for oocyte retrieval. The key is to aim for a pharmacological exposure of shortest duration.

### Use of Anaesthetic Drug

While selecting a desired agent our main concerns are:-

- Whether the substance enters the follicular fluid?
- What are its toxic effects on the fertilization and clevage and pregnancy rates?

### Drugs commonly used

**• Propofol**

Widely being used in assisted reproduction and its effects on the fertilization, embryo clevage and pregnancy rates has been extensively studied. Propofol has added advantages of antiemetic property along with faster recovery.

Though earlier studies had documented adverse effects of increased exposure to propofol on clevage of oocytes^17^,^18^, a recent study showed that although propofol follicular concentration increases with time, there was no difference in the rates of mature to immature oocytes^19^.

In addition, there was no significant difference found in fertilization rate, clevage and embryo cell number, implantation rate as compared to thiop entone. Except a trend towards low fertilization rate with longer exposure to anaesthetic drug^19^,^20^.

**• Role of Nitrous – Oxide**

**Its role still remains controversial.** Gonen etal found out that nitrous oxide has deleterious effect on IVF outcome^21^ N_2_O inactivates methionine synthetase thereby decreasing the amount of thymidine available for DNA synthesis in dividing cells. However, as the inactivation of methionine proceeds slowly in the human liver, the effect of N_2_O is minimal. Further more, the low solubility of N_2_O exposes the oocytes to this gas for a brief duration. Rosen etal in 1987 found no significant difference between the fertilization or pregnancy rates when comparing isoflurane with O_2_ which was further confirmed by Matt et al^22^,^23^

While, Hadimioglu N et al in 2002 showed nitrous oxide actually increase the rate of IVF by reducing the concentration of other potentially toxic and less diffusible anaesthetic drugs^14^.

**• Benzodiazepine**

Midazolam is the most commonly used benzodiazepine. Although minimal amount of this benzodiazipine are found in follicular fluid, no detrimental effects have been proven so far^24^. A combination of midazolam and fentanyl was found to be safe for oocyte retrieval^5^,^25^.

**• Narcotics**

In recent years, various opioids have been used as a part of regime in conscious sedation and monitored care for anesthesia in assisted reproductive technique.

Fentanyl or alfentanil were found to be favourable agents when used in combination with propofol by Hadimioglu etal in 2002. Fentanyl has minimal penetration into follicular fluid^26^,^27^. Alfentanil follicular fluid level is 10 fold smaller than the serum concentration at the same point^28^.

**• Ketamine**

A randomized prospective study, found the combination of midazolam and ketamine a good alternative to general anaesthesia^29^.

### Drugs to be avoided

### Inhalational Agents

Majority of studies have shown detrimental effect of halogenated fluorocarbons with N_2_O resulting in decreased clevage rates and increased abortions^30^.

Matt etal in 1991 found no significant effect of N_2_O and isoflurane anaesthesia on human IVF pregnancy rate^22^.

### Use of Regional Anaesthesia

1. It constitutes either central neuraxial blockade or the peripheral nueral block.
  - Paracervical block with different doses of lidocaine with sedation has been used by anaesthetist for egg retrieval^31^‐^34^. Corson etal have even used paracervical block with bupivacaine for pain relief during oocyte aspiration^35^. Various conscious sedation regimens using midazolam, diazepam, alfentanyl have been used along with paracervical block to enhance the analgesia^32^. Electroacupunture has also been used with paracervical block to improve the effectiveness of pain relief^36^.
  - Spinal anaesthesia is also an effective method. Martin et al in 1998 had used low dose hyperbaric 1.5% lidocaine (45mg) spinal with low dose fentanyl 10mcg for egg retrieval^37^. Tsen had compared low dose bupivacaine +fentanyl with lidocaine+fentanyl for oocyte retrieval and did not find any combination superior to other^38^
  - Epidural anaesthesia also forms a viable option but does not demonstrate any advantage over intravenous sedation^39^.
2. Bupivacaine compared favorably to lidocaine in all aspect except taking approximately 30 min longer to micturition and to discharge^40^.
3. Hormonal response to follicular puncture is fully attenuated by regional anaesthesia and partially by technique requiring sedation^41^.

### Alternative Therapy: Acupunture

It is a traditional Chinese medicine, nontoxic, relatively affordable, therapy with possible indications as an adjunct in assited reproduction with the following beneficial effects:

1. Sympathoinhibitory
2. Increased beta-endorphin levels
3. Antidepressant, anxiolytic
4. Neuroendocrine effect on hypothalamic – pituatry-ovarian axis
5. Increased uterine blood flow

Electroacupuneture has been used with along with paracervical block for analgesia during oocyte retrieval^42^. Various conscious sedation regimens have been used along with electroacupuncture to enahance analgesia for oocyte retrieval^43^‐^45^.

The technique employed in aspiration of the oocyte and laborotry manipulations have all been modified and updated. Which is better, sedation or general anaesthesia is more of a personal preference. But the anaesthetic which is important to the comfort level both for the patient and the gynaecologist to maximize the harvesting of oocytes plays an important role in the successful outcome.

**How Safe are Anaesthetic Agents?** With the coming up of large prospective trials documenting safe use of drugs like propofol, opioid, the newer anaesthetics have lost their inhibitions regarding the use of these agents, thereby widening the scope of more rationale anaesthesia in IVF and extending our services to this developing sub-speciality.

The key to anaesthesia in IVF is to aim for pharmacological exposure of shortest duration with minimal penetration to follicular fluid.
